# Quality of Bowel Preparation in the General Population

**DOI:** 10.3390/medicina62010063

**Published:** 2025-12-28

**Authors:** Melanija Ražov Radas, Ivo Klarin, Marija Ljubičić

**Affiliations:** 1 Department of Gastroenterology, Internal Medicine Clinic, Zadar General Hospital, 23000 Zadar, Croatia; klarin.ivo@gmail.com; 2 Department of Health Studies, University of Zadar, 23000 Zadar, Croatia; mljubicic@unizd.hr

**Keywords:** colonoscopy, bowel preparation, polyethylene glycol, colorectal carcinoma

## Abstract

*Background and Objectives*: Colorectal cancer caused over 1.9 million new cases and 0.9 million deaths in 2020, ranking third in incidence and second in cancer mortality worldwide. Poor bowel preparation may hide adenomas, increasing the colorectal cancer risk. This retrospective study aims to identify differences and associations in bowel preparation quality in relation to gender, age, timing of preparation, and the type of cleansing agent used. *Materials and Methods*: We analyzed the quality of bowel preparation in a total of 4609 colonoscopies performed between June 2019 and April 2022. We used *t*-tests and ANOVA to assess differences in bowel preparation quality according to participants’ characteristics. The multivariable linear and logistic regression analyses were used to evaluate the association between bowel preparation quality, withdrawal time, adequate bowel preparation, and total colonoscopy. *Results*: 70.9% of patients had adequate bowel preparation quality. Enema (β = −0.20, *p* < 0.001), bisacodyl (β = −0.16, *p* < 0.001), and senna solution (β = −0.03, *p* = 0.012) were linked to poorer bowel preparation quality in comparison with polyethylene glycol. Older age was associated with a slight decrease in the probability of adequate bowel preparation (adjusted OR 0.98 per year, *p* < 0.001), whereas female gender was associated with an increase in this probability (OR 1.18, *p* = 0.038). Bowel preparation in winter is associated with a lower likelihood of adequate preparation compared to summer (OR 0.74, *p* = 0.004). The type of bowel preparation agent affected outcomes, with enema (OR 0.22, *p* < 0.001) and bisacodyl (OR 0.35, *p* < 0.001) associated with the less clean bowels. Longer withdrawal time was inversely associated with adequate preparation (OR 0.94, *p* < 0.001). For total colonoscopy, the strongest predictor was adequate bowel preparation (OR 23.6, *p* < 0.001), with gender, ulcerative colitis, and polyps also influencing the outcome. *Conclusions*: Age, gender, season, medications, withdrawal time, and the type of colorectal lesions influence bowel preparation quality. Personalized approaches, including patient education and targeted interventions, might contribute to improved bowel preparation, especially in older patients, and should be evaluated in future studies.

## 1. Introduction

Colorectal cancer (CRC) accounted for over 1.9 million new cases and approximately 0.9 million deaths in 2020, making it the third most common cancer and the second leading cause of cancer-related mortality worldwide [1]. The incidence of CRC is constantly increasing, but with the introduction of a screening program with adequate bowel preparation, it is expected that the disease will be detected at an early stage, leading to a decrease in the number of patients who, at the time of diagnosis, are in the terminal phase of the disease. In Europe, in 2022, according to the European Cancer Information System (ECIS), there were an estimated 361,986 new cases of colorectal cancer [2]. Among them, post-colonoscopy, colorectal cancer (PCCRC) accounts for 3.6% to 9.3%, making it a significant clinical concern [3]. More than 80% of PCCRC cases can be prevented [3].

Colonoscopy is the gold standard for CRC screening, but its accuracy is related to the high quality of bowel preparation. The quality of colonoscopy is assessed using objective measures such as the adenoma detection rate (ADR) and cecal intubation success, with both endoscopist skill and adequate bowel preparation being crucial [4]. The guidelines define adequate bowel preparation as the ability of the endoscopist to identify polyps greater than 5 mm [5,6]. A ≥90% minimum standard for adequate bowel preparation (assessed using validated scales) has been recommended (2019) by the Quality Committee of the European Society of Gastrointestinal Endoscopy (ESGE) [5]. However, nearly one in four procedures is still performed with the suboptimal bowel preparation, leading to longer procedure times, a higher risk of complications, and an increased likelihood of missed lesions [7]. Adenoma detection rate as a precursor to the formation of CRC is directly related to the degree of bowel preparation for colonoscopy [8]. Therefore, it is essential to evaluate performance metrics, including quality of bowel preparation, cecal intubation rate, adenoma detection rate, and withdrawal time, as well as the incidence of PCCRC and incomplete resections at all levels [3,5].

Bowel cleansing is a key component of a high-quality colonoscopy. There is a wide range of multicausal factors that result in inadequate bowel preparation [9]. Despite recent advances, not all predictors of successful cleansing are fully understood, and their roles are variably reported in the literature [10]. A meta-analysis of 125 studies reports that patient-related determinants include older age, male sex, diabetes, chronic constipation, and use of medications such as the opioids or tricyclic antidepressants [9]. The risk factors for inadequate preparation can be summarized into a few main categories: demographic variables, comorbidities, medications, the preparation process, and indications for colonoscopy [9]. Other studies have also noted that age, duration of bowel preparation, and laxative dose are factors that affect the adequacy of the bowel preparation and patient compliance [11]. The age is associated with lower completion rates and a higher likelihood of poor bowel preparation [12]. Elderly patients or those with significant comorbidities are more likely to have inadequate bowel preparation [13]. Colonoscopy in very elderly patients carries a greater risk of complications and morbidity than in younger patients [11,14]. Moreover, differences in bowel preparation quality between men and women have been observed, suggesting that gender, in specific approaches, might enhance cleansing success [10]. Studies show that females achieve better bowel preparation than males [14,15].

Procedural factors such as the type and timing of the bowel cleansing regimen also play a role [16,17]. On the other hand, some studies highlight that the type of bowel preparation did not affect the procedure duration or the quality of visualization [13]. Seasonal variations have also been reported. A large retrospective cohort study found that the season of colonoscopy (e.g., spring vs. winter) significantly affects the cleansing quality [14].

Several comorbid conditions, such as diabetes mellitus, chronic liver disease (cirrhosis), neurological disorders (Parkinson’s disease, dementia), reduced mobility, prior abdominal surgery, and chronic constipation, have been identified as independent predictors of inadequate bowel preparation [9,18]. These conditions may impair colonic motility, delay intestinal transit, or reduce the patient’s ability to comply with preparation instructions. In addition, although diverticulosis and anorectal conditions such as hemorrhoids are common, several studies have suggested that the presence of diverticula or colorectal tumors may be associated with lower quality bowel preparation [19].

In addition, hospitalization, poor adherence to dietary instructions, cognitive function, low health literacy, and adherence can contribute to suboptimal preparation [20,21]. Low adherence to dietary restrictions (high-residue food) contributes to poor bowel preparation, while the use of instruction of bowel preparation is associated with better quality [5].

Inadequate bowel preparation can not only increase the risk of missed early colorectal lesions and, most importantly, adenomas (the highest potential to progress to colorectal cancer), but also leads to patient fatigue and the need for repeat procedures. ESGE recommends repeating the colonoscopy within one year following inadequate bowel preparation [5], which makes the procedure more expensive and increases the cost of the waiting list [22].

Given these multifactorial determinants, this study contributes to the existing literature by providing additional insight into how demographic factors (age, gender), seasonal factors, and bowel preparation–related factors influence preparation quality [10,11,13,14]. Additionally, it sheds light on procedural factors, such as withdrawal time, and patient-related factors, including gender and the presence of previously diagnosed bowel disease. Another important factor is the completion of total colonoscopy, which is often not achieved due to inadequate preparation. By examining these associations, the study contributes to clarifying aspects that have been less thoroughly explored or where previous studies have reported conflicting results. This provides insights that may help optimize colonoscopy preparation and improve clinical practice.

This study aimed to identify factors (age, sex, indication, season, timing, and type of preparation) associated with the bowel preparation quality in a general population undergoing screening and diagnostic colonoscopies. We hypothesized that people’s different activities and food habits in different seasons contribute to the quality of the bowel preparation varies in different seasons. Also, we hypothesized that gender is not an independent risk factor for quality in bowel preparation, but that age is. The quality of bowel preparation varies according to gender, age, timing of preparation, and the type of cleansing agent used. The use of certain bowel cleansing agents affects the quality of bowel preparation, whereas adequate bowel preparation positively correlates with higher BBPS scores and increases the likelihood of completing a full colonoscopy.

## 2. Materials and Methods

### 2.1. Participants

We performed a total of 4835 colonoscopies from June 2019 to April 2022, using Pentax colonoscopes of different generations. A retrospective descriptive analysis was performed on the clinical records of all patients (inpatient, outpatient without repeated colonoscopies) who met the inclusion criteria. Inclusion criteria were age > 18, indicated for colonoscopy from June 2019 to April 2022 (during the COVID-19 pandemic). We excluded patients with contraindications for colonoscopy: pregnancy, intestinal occlusion, and perforation or threatening perforation (severe inflammation, etc.). We excluded 231 patients who did not meet the inclusion criteria, namely 162 with an undefined cleansing agent, 7 with an undefined extent of examination, 45 without documented withdrawal time, and 17 procedures performed by physicians with a low number of colonoscopies (insufficient experience to be considered proficient). The final number of colonoscopies analyzed was 4603.

The data come from the hospital’s electronic medical record system, including age, sex, the indication of colonoscopy, risk factors for colonoscopy, day of the week, conscious sedation (pethidine, midazolam) administered by gastroenterology-trained nurse, or propofol sedation administered by an anesthesiologist with or without an anesthesia assistant, diagnosis (irritable bowel disease, tumors, polyps, diverticula, anorectal changes), total colonoscopy time, withdrawal time, and Boston bowel preparation score (BBPS). Endoscopists with a minimum of five years of experience performed the examination and assessed the cleanliness according to the BBPS scale.

### 2.2. Ethical Consideration

This study was approved by the Ethics Committee of the Zadar General Hospital (No. 01-2529/25-9/25, from 31 March 2025). All patients signed an informed consent for a colonoscopy before the exam. Following relevant ethical guidelines, all patient data were anonymized before analysis to protect privacy and confidentiality. As it is a retrospective analysis of existing clinical de-identified data, informed consent of the participants was not required for participation. The study complied with the Declaration of Helsinki and laws regulating the use of retrospective data in clinical research.

### 2.3. Protocol of Bowel Preparation

The patients were advised to avoid fruits, vegetables, seeds, and a high-residue diet for five days before the procedure, and to take a bowel cleansing solution after a light lunch the day before the colonoscopy. A split-dose regimen was recommended. For bowel preparation, patients were offered two different solutions: Solution 1: solution of macrogol 3350 (polyethylene glycol), sodium sulfate, sodium chloride + potassium chloride + sodium ascorbate, and ascorbic acid (PEG-Original name: Movi Prep); Solution 2: bisacodyl + magnesium solution in an amount of 3–4 L. The patients who were examined in an emergency or who were not able to drink a cleansing agent (uncooperative, seriously ill, or patients with active rectal bleeding) were examined without using enemas.

The Boston Bowel Preparation Scale (BBPS), which has been validated in multiple clinical studies, was used as a basic tool for determining the degree of bowel cleanliness [2,3]. The Boston Bowel Preparation Scale was used to assess the success of bowel preparation, grading each colon segment from 0 to 3 to indicate the level of cleanliness during colonoscopy (0 = the colon segment is unprepared; the mucosa is completely obscured by solid stool, and the fecal material cannot be cleared; 1 = parts of the mucosa are visible, but other areas remain poorly seen due to stool or opaque liquid obstructing the view; 2 = the mucosa is largely visible, with only small amounts of residual stool, fragments of feces, or opaque liquid; 3 = the entire mucosa is clearly visible, with no remaining stool or soiling) (2,3). BBP Score was present as excellent (BBPS 8–9), good (BBPS 6–7), average (BBPS 4–5), or poor (BBPS 0–3) (2,3). The BBPS should be presented in the examination report using the following documentation: right/transverse/left; e.g., = 2/2/3, along with the corresponding total (BBPS = 7). Poorer adenoma detection rate is in correlation with poor bowel preparation (BBPS ≤ 5) [5]. The overall BBPS score was classified as adequate (≥6) or inadequate (0–5) [2,3,5,7].

### 2.4. Data Analysis

The statistical analysis was conducted using Statistica 13 (TIBCO Software Inc., San Ramon, CA, USA, 2017) on total sample of 4609 Croatian respondents. The statistical significance was *p* < 0.05.

In the descriptive analysis, mean and standard deviation were used for numerical variables, while absolute numbers and percentages were used for categorical variables. In the bivariate analyses, the chi-square test was used to examine the differences between variables for categorical variables, while the Student’s *t*-test and ANOVA were used for numerical variables. Significant Bonferroni-adjusted differences are reported where applicable. The Pearson correlation coefficient was used to examine the relationship between variables.

The multivariate analysis included linear and logistic regression to assess the relationship between the variables. In the linear regression models, the quality of bowel preparation using the BBPS scale and withdrawal time were used as dependent variables. The predictors for these two models were age, gender, indication for colonoscopy (preventive and control examinations were the referent group, RG), risk factors such as comorbidities, anticoagulant therapy, and age over 80 years. (No risk was the RG), season (summer was RG), the timing of bowel preparation (day before the examination was RG), bowel cleansing procedure (the working day was RG), bowel preparation medication (PEG solution was the reference group), the premedication or sedation (no was RG), irritable bowel disease, tumor, polyp, diverticulum, anorectal changes, and adequate bowel preparation (no was RG). Logistic regression models were used to predict the probability of adequate bowel preparation and complete colonoscopy. The prior regression model described the predictors and the reference groups, while beta was converted to OR (odds ratio) with a 95% confidence interval. To prevent bias, the variables included in the multivariable linear and logistic regression models were selected based on clinical relevance and the literature. All selected variables were entered simultaneously into the models. The multicollinearity among the independent variables was assessed using variance inflation factors, and all values ranged between 1 and 2, indicating the absence of the significant multicollinearity. Potential interactions among the variables were also tested, and none were statistically significant.

## 3. Results

### 3.1. Characteristics of the Study Group

A total of 4609 patients were included in this study. The average age of the respondents was 62 (M = 62; SD = 14.0). Respondents aged between 61 and 75 were the most strongly represented, with a total of 43.9% (Table 1). Women (53.7%) and men (46.3%) were approximately equally represented in the sample. The most common indications for colonoscopy were symptomatic (53.2%). The respondents were mostly risk-free for colonoscopy (96.3%) and without sedation (94.4%). A certain percentage of subjects had irritable bowel disease (4.4%), tumors (4.8%), polyps (22.9%), diverticula (24.8%), and anorectal changes (59.8%) (Table 1).

The respondents were mainly examined in winter (30.6%) and spring (29.1%). The number of colonoscopies was evenly distributed across the days of the week. Bowel preparation was carried out the day before the examination (93.4%), with solution 1 (PEG).

Overall, 70.9% of patients had adequate bowel preparation quality (BBPS ≥ 6). The overall quality of bowel preparation (BPPS score) was 6.2, while the average quality of colon preparation ranged from 1.7 for the right colon, 2.1 for the transverse colon, and 2.4 for the left colon. Quality of bowel preparation was adequate (BBPS ≥ 6) in 3268 (70.9%) patients, while it was inadequate (BBPS ≤ 5) in 1341 (29.1%) patients.

Bowel preparation was excellent in (BBPS 8–9) 1473 (32.0%), good (BBPS 6–7) in 1795 (38.9%), poor (BBPS 4–5) in 715 (15.5%), and inadequate (BBPS 0–3) in 626 (13.6%) patients. The withdrawal time was 8.7 ± 2.7 min, while the complete colonoscopy was performed in 87.9% of the subjects (Table 1).

### 3.2. Differences in the Bowel Preparation Quality According to Respondents’ Characteristics

The differences by age group, gender, season, day of the week, and bowel preparation solution in the category of quality of bowel are shown in Supplementary Table S1. Differences in bowel preparation quality according to respondents’ characteristics are also shown (Table 2).

Younger adults had higher scores (*p* < 0.001). Subjects who underwent colonoscopy in summer had a better degree of quality of bowel preparation (6.4 ± 2.1), while they were least prepared in winter (6.1 ± 2.0), *p* = 0.002. Respondents with the colonoscopy performed on Thursday had the best bowel preparation score (6.4 ± 2.0) and the lowest on Friday (6.0 ± 2.0), *p* = 0.001. Respondents who had performed bowel preparation on Sunday had a higher degree of bowel preparation (6.4 ± 2.0) than on weekdays (6.2 ± 2.1), *p* = 0.008 (Table 2).

Subjects with a preventive indication for colonoscopy showed a statistically significantly better degree of bowel preparation (6.6 ± 1.8) compared to symptomatic (6.1 ± 2.1) and control subjects (6.2 ± 2.0), *p* < 0.001. PEG showed the best bowel preparation effect compared to the other preparations (6.4 ± 1.9), while the enema showed the worst results (3.9 ± 2.3), *p* < 0.001 (Table 2).

Subjects who received conscious sedation (pethidine, midazolam) administered by the gastroenterology-trained nurse or propofol sedation administered by an anesthesiologist had the highest quality of bowel preparation (7.2 ± 1.7), *p* < 0.001). Subjects with disease (7.2 ± 1.8) had significantly better bowel preparation quality, while patients with tumors had poorer (5.2 ± 1.7), *p* < 0.001. No statistically significant difference in bowel preparation was found between subjects with and without polyps (*p* = 0.105). Subjects with hemorrhoids and other anorectal changes had statistically significantly higher quality of the bowel preparation (6.4 ± 1.9) than subjects without hemorrhoids (5.9 ± 2.2), *p* < 0.001.

Subjects with adequate bowel preparation had a significantly higher degree of cleanliness (7.3 ± 1.1) than subjects with inadequate bowel preparation (3.6 ± 1.3), *p* < 0.001. Subjects with complete colonoscopy had better bowel preparation (6.6 ± 1.7) when compared to those with incomplete colonoscopy (3.1 ± 1.7), *p* < 0.001 (Table 2).

Significant differences after Bonferroni correction are indicated in Table 2.

### 3.3. Relationship Between the Quality of the Bowel Preparation and Characteristics of the Study Group

The linear regression model confirms that older respondents have a lower quality of bowel preparation (β = −0.12, *p* < 0.001). Respondents who had a colonoscopy in summer had a better quality of bowel preparation when compared to winter (β = −0.07, *p* < 0.001), and spring (β = −0.06, *p* = 0.001). The linear regression model confirms that Senna solution (β = −0.03, *p* = 0.012), Enema (β = −0.20, *p* < 0.001), and Bisacodyl (β = −0.16, *p* < 0.001) showed a lower impact on the quality of bowel preparation compared to PEG (Table 3).

A better quality of bowel preparation was found in subjects who received premedication (β = 0.09, *p* < 0.001), in subjects with Crohn’s disease (β = 0.06, *p* < 0.001), diverticulitis (β = 0.06, *p* = 0.043), and anorectal changes (β = 0.10, *p* < 0.001), while those with a tumor were associated with poor quality of bowel preparation (β = −0.06, *p* = 0.010). There was no association between polyposis and quality of bowel preparation (β = −0.02, *p* = 0.145).

Longer withdrawal time is positively associated with the symptomatic indication for colonoscopy (β = 0.06, *p* < 0.001), spring (β = 0.06, *p* < 0.001), bowel preparation on Sunday (β = 0.04, *p* = 0.002), and polyposis (β = 0.12, *p* < 0.001). A negative association was found with bisacodyl (β = −0.08, *p* < 0.001), ulcerative colitis (β = −0.03, *p* = 0.026), and anorectal changes (β = −0.19, *p* < 0.001).

A longer withdrawal time was associated with a better quality of bowel preparation (β = 0.09, *p* < 0.001) (Table 3).

Compared to younger respondents, older respondents had a lower likelihood of having adequate bowel preparation (odds ratio (OR) = 0.98, 95% confidence interval (CI) = 0.97–0.99, *p* < 0.001). Each additional year of participants’ age slightly reduced the probability of adequate bowel preparation. In addition, respondents who prepared in winter had less likelihood of having adequate bowel preparation (OR = 0.74; 95% CI = 0.60–0.91; *p* = 0.004) than those who prepared in summer. The bowel preparation in winter reduces the likelihood of adequate preparation compared to summer. Compared to PEG, other solutions had a lower likelihood of adequate bowel preparation (Senna solution (OR = 0.57; 95% CI = 0.34–0.96; *p* = 0.035), enema (OR = 0.22; 95% CI = 0.14–0.36; *p* < 0.001), bisacodyl (OR = 0.35; 95% CI = 0.20–0.61; *p* < 0.001). Longer withdrawal time is associated with lower odds of adequate bowel preparation (OR = 0.94; 95% CI = 0.92–0.97; *p* < 0.001), i.e., more time procedures when the bowel preparation is suboptimal (Table 4).

Lower odds of total colonoscopy were associated with older age (OR = 0.99; 95% CI = 0.98–1.00; *p* = 0.041), female (OR = 0.77; 95% CI = 0.57–0.90; *p* = 0.004), symptomatic indication (OR = 0.54; 95% CI = 0.42–0.68; *p* < 0.001), patient with enema (OR = 0.36; 95% CI = 0.22–0.58; *p* < 0.001), bisacodyl (OR = 0.24; 95% CI = 0.13–0.45; *p* < 0.001), and tumor (OR = 0.22; 95% CI = 0.14–0.34; *p* < 0.001). Higher odds for total colonoscopy associated with polyposis (OR = 1.43; 95% CI = 1.06–1.91; *p* = 0.018) and with longer withdrawal time (OR = 1.27; 95% CI = 1.22–1.33; *p* < 0.001).

The odds ratio for total colonoscopy was OR = 23.60; 95% CI = 17.84–31.22; *p* < 0.001, indicating that patients with adequate bowel preparation were 23.6 times more likely to have a total colonoscopy than those with inadequate bowel preparation (Table 4).

## 4. Discussion

The objective of this study was to assess the validity and reliability of existing bowel preparation solutions regarding age, sex, indication, season, and day of the week of preparation for colonoscopy. Our results show that older age was associated with a slight decrease in the probability of adequate bowel preparation, meaning that each additional year of age slightly reduced the chances of adequate preparation. The female gender was associated with adequate preparation, while the type of bowel preparation agent had a significant effect, with some agents substantially reducing the chances of adequate preparation. Premedication or sedation was associated with adequate preparation, while the presence of diverticula and anorectal changes also contributed to a higher probability of adequate preparation. A longer withdrawal time had a smaller but still significant effect, likely reflecting the suboptimal bowel preparation. Therefore, endoscopists need to spend more time examining poorly prepared colons. This not only prolongs the procedure but may also increase patient discomfort, emphasizing the importance of adequate bowel preparation before colonoscopy. For complete colonoscopy, the predictor was adequate bowel preparation, while gender, the presence of ulcerative colitis, or polyps also influenced the outcome. Other variables, such as season, Morbus Crohn, and age groups, did not have a significant effect. Compared to younger respondents, older respondents had a lower likelihood of adequate bowel preparation. In addition, respondents who were prepared in summer had better BBPS scores than in winter. Compared to polyethylene glycol, other solutions had a lower likelihood of adequate bowel preparation. To achieve adequate bowel preparation, a conversation with the patient is required, with a thorough interpretation of the need to follow the dietary instructions and the need to drink the cleansing solution in an adequate and at an adequate manner.

Zadar General Hospital serves approximately 170,000 patients, representing 4.85% of the total population of the Republic of Croatia (about 3.5 million). In line with scientific recommendations, the country has implemented a national colorectal cancer screening program aimed at the early detection of premalignant and malignant lesions when the disease is still curable. Patients undergo colonoscopy for various indications, including participation in the national screening program, the presence of different symptoms, preventive procedures, and follow-up colonoscopies. This diverse patient population is important as it provides a representative sample for evaluating factors that influence the bowel preparation quality and the effectiveness of colonoscopy in real-world clinical practice.

Our results show a low rate of adequate bowel preparation for colonoscopy, with most participants being older adults: over 75% were older than 45 years, and nearly 3000 participants were over 60 years of age. This age distribution significantly contributes to poorer preparation, which is consistent with previous studies indicating that older individuals are at higher risk for inadequate preparation due to reduced gastrointestinal motility, comorbidities, polypharmacy, and potential difficulties in following instructions [9,18,23].

We observed several discrepancies compared with previously published evidence on factors influencing the quality of the bowel preparation. Although the literature often reports gender-related differences in preparation quality, our study partially confirms such an association [11,14]. A study by Shi et al. (2023) identified male gender, inpatient status, and the spring season as independent risk factors for inadequate bowel preparation [14]. In contrast, our study found that female gender was associated with a higher likelihood of the adequate bowel preparation, aligning with the results reported in previous studies [10,14]. Although no significant difference in mean BBPS scores was observed between genders in the bivariate analysis, logistic regression analysis indicated that female patients have a higher probability of achieving adequate bowel preparation, showing a clinically important difference that is not evident when looking at average scores. Similarly, while the impact of seasonal variations is less frequently addressed in prior research, our results indicated a pattern suggesting better bowel preparation during the summer months, with the lowest quality observed in winter. This pattern may imply that seasonal factors influence patients’ adherence or physiological responses relevant to bowel cleansing, which could be explored in future studies [14].

The participants who completed their preparation during the winter period were also less likely to achieve adequate bowel cleansing, further supporting the possibility of seasonal differences. However, these results should be interpreted with caution, as no clear or definitive explanation can be provided. Seasonal fluctuations in dietary habits, physical activity, or daily routines may vary across populations and cultural contexts, though such assumptions should not be overstated without data on participants’ nutritional patterns [14]. Overall, these findings highlight the need for further research to explore the potential influence of seasonality on bowel preparation quality and to clarify the mechanisms that may underlie the differences observed in this study.

Compared to polyethylene glycol (PEG), other solutions had a lower probability of adequate bowel preparation [24]. In our study, linear regression showed that PEG was statistically significantly better compared to other preparations. Namely, both linear regression and odds ratio analyses in our study consistently showed that PEG was associated with the better bowel preparation quality, whereas enema and bisacodyl were linked to poorer outcomes. This is supported by randomized studies showing that adding an enema to standard bowel preparation does not improve colon cleansing and may reduce patient acceptability, suggesting that enemas are less effective than PEG-based regimens [5]. Another study noticed that the type of volume (high volume: 4 L polyethylene glycol or low volume: 2 L polyethylene glycol + bisacodyl) did not by itself significantly affect bowel preparation quality, but the method of administration and adherence to the regimen may serve as predictors of success [25]. Therefore, the Quality Committee of the European Society of Gastrointestinal Endoscopy does not recommend routine use of enemas in bowel preparation [5].

Furthermore, this and other studies highlight that bowel preparation outcomes are influenced by a combination of patient-related factors, such as age, gender, and comorbidities, and the characteristics of the preparation regimen itself [10,11,13,14,25].

Studies confirm that age was a key factor in predicting poor bowel preparation in hospitalized patients [11]. For every 10-year increase in age, the odds of having poor bowel preparation increased by 1.29 [23]. In our study, compared to younger respondents, older respondents had a lower likelihood of having adequate bowel preparation. Each year increase in age of the participants slightly decreased the odds of adequate bowel preparation. The subjects under 45 years of age showed a better level of quality of bowel preparation than older subjects. The older subjects had a lower level of bowel preparation quality. Colonoscopy in older subjects carries a greater risk of complications and morbidity than in younger patients and is associated with lower completion rates and a higher likelihood of poor bowel preparation [14,23].

There is still plenty of room for improvement in results. It is unusual that sedation was associated with better prep. This result can be explained by the small number of sedated patients (only 5.3%), but also by the fact that in the sedated group were younger patients, most often suffering from inflammatory bowel disease [26]. Propofol or conscious sedation led to no difference in colonoscopy-related quality metrics [27]. In our study, subjects who received conscious sedation (pethidine, midazolam) administered by a Gastroenterology-trained nurse or propofol sedation administered by an anesthesiologist had the highest quality of bowel preparation. However, since sedation is administered only after the completion of the bowel preparation process, the observed association between sedation and preparation does not imply that sedation improves preparation. This association is likely the result of other factors influencing the course and quality of the preparation process.

Monday had the highest rate of inadequate preparation (BBPS < 6) compared to other days of the week [7,28,29]. Post-holiday procedures were not associated with poor bowel preparation [28,29]. In our study, patients with the colonoscopy performed on Thursday had the best bowel preparation score and the lowest on Friday. Patients who performed bowel preparation on Sunday had a higher degree of bowel preparation than on weekdays. It is possible that patients do not follow the instructions on the diet protocol during the work week due to a busy lifestyle.

To summarize, older age and off-peak season (winter) predict poorer prep, and the PEG solution outperforms others. Hence, preparation during summer and recommendations for using the PEG solution may give better results. These align with previous reports (age is often a factor). Overall, 70.9% of patients had adequate bowel preparation quality (BBPS ≥ 6), which is unsatisfactory. Guidelines aim for an adequate bowel preparation quality rate of more than 85% [5]. This finding highlights the need for improved bowel preparation protocols and better patient education. Better communication between patients and staff and awareness campaigns may contribute to improvement, additional measures, such as split-dose regimens or low-residue diets, could further enhance preparation quality [5].

We observed that patients with diverticula and anorectal changes demonstrated better bowel preparation for colonoscopy. This may be explained by their higher motivation for thorough cleansing and frequent experiences with gastrointestinal symptoms. Since they generally adhere to dietary instructions and doctors’ advice, patients with diverticulosis are more likely to follow the bowel preparation instructions more effectively [30]. However, other studies indicate an association between the presence of diverticula and inadequate bowel preparation [19]. In our study, the presence of symptoms, tumor, or polyp was not associated with the adequacy of the bowel preparation, but it was linked to completion of total colonoscopy. Based on available data, we did not identify any studies explicitly showing that the presence of colorectal tumors independently reduces the quality of bowel preparation. It is possible that, due to awareness of the potential malignancy, the endoscopists or the patients made extra efforts to ensure the procedure reached the cecum, thereby avoiding incomplete examination and preventing any potential worsening of a malignant condition. Previous colonoscopy experiences and enhanced counseling by physicians may also contribute [31]. Additionally, greater health awareness and adherence to dietary or pharmacological regimens before the procedure could play a role [32]. This is supported by meta-analyses of 125 studies, which show that stool consistency, incomplete preparation intake, and noncompliance with dietary instructions are strong predictors of inadequate bowel preparation [9].

In our study, overall, 70.9% of patients had the adequate bowel preparation quality (BBPS ≥ 6). However, adequately prepared bowel is the first step in reliably detecting small lesions and distinguishing adenomas from lesions that need to be resected [3]. This is especially important for lesions in the left colon. According to Kaminski key performance measures, minimum targets are rate of the adequate bowel preparation of ≥90% and cecal intubation rate of ≥90% [33].

A lot of effort is needed to improve the results of bowel preparation. In an era where digitalization and artificial intelligence are making a big entrance into colonoscopy, an AI-assisted approach could help identify suboptimal bowel preparation in real time, develop support systems, and provide personalized feedback to patients to optimize cleansing outcomes [3]. Recent studies suggest that the use of artificial intelligence (AI) driven tools can help optimize bowel preparation. AI applications can provide real-time guidance and personalized recommendations, improving patient adherence and overall quality of bowel cleansing [34].

The medical staff may use a short video presentation on adequate bowel preparation. The patient remembers a visual representation better than only a written document with instructions for prep. Visual reminders of a low-residue diet are also a good choice. Also, a few days before the procedure, a text message to remind patients of their prep schedule and instructions can be sent. Implementing a smartphone app can substantially improve the adequacy of the bowel cleansing and patients’ compliance with preparation instructions [34].

To thoroughly and completely examine the colon, it is necessary to cooperate with the patient and be maximally engaged in preparing the intestine before the examination. For better bowel preparation and a thorough, complete examination of the colon, it is essential to ensure patient cooperation and to be fully engaged in preparing the bowel prior to the procedure [35]. Better cooperation between patients and medical staff is needed, as are public health campaigns to raise awareness of the importance of proper preparation for colonoscopy. The findings of the present study need to be validated by multicenter prospective studies that include more information about patients’ medical histories. In patients with risk factors for inadequate bowel preparation, enhanced preparation and clear instructions can help improve the quality of bowel cleansing [14,35].

The distinctive feature of this study is that it was conducted during the COVID-19 pandemic. Despite resource constraints related to the pandemic, increased oversight, and prioritization measures associated with COVID-19, colonoscopy rates remained approximately stable during the COVID period compared with the pre-COVID period [35]. Recent studies confirm that although colorectal cancer screening rates declined during the COVID-19 pandemic, by 2023 they had returned to levels equal to or even higher than those before the pandemic [36]. However, to our knowledge, no studies have specifically compared demographic characteristics and other risk factors, or bowel preparation adequacy, before, during, and after the COVID period, highlighting the need for new studies to address this gap.

It is particularly important to identify high-risk patients. By applying predictive models, it is possible to identify individuals at increased risk of inadequate bowel preparation in advance [37,38]. This allows for a personalized preparation approach, reduces the need for repeat colonoscopies, enhances diagnostic efficacy, and optimizes healthcare resources. Improving patient cooperation through visual aids and reminders, along with public health campaigns, can enhance bowel preparation quality for colonoscopy. High-quality bowel preparation also provides a significant cost–benefit effect. It decreases the burden on the healthcare system both by reducing waiting lists and by contributing to the prevention of malignant diseases, but these effects should be verified in future studies [22].

There are some limitations of this study. First, its single-center and retrospective design may limit the generalizability of the findings. Second, we did not stratify analyses by inpatient versus outpatient status, which could influence the bowel preparation quality and colonoscopy outcomes. Patients’ education and history of colon preparation, comorbidities such as chronic cardiac failure, antidepressant drugs, diabetes mellitus, etc., were not recorded in this study. Additionally, retrospective data may have missed unmeasured confounders, and dietary instructions were not standardized or verified, potentially affecting preparation quality. Finally, because bowel-preparatory methods were not randomly assigned, there is a possibility of selection bias. However, the large sample size (over 4500) is a strength of this study, together with a randomly selected patient preparation protocol. The use of the BBPS provides a standardized method for assessing the adequacy of bowel preparation. Additionally, patient education has been shown to improve overall the bowel cleansing quality, highlighting the importance of clear instructions and professional guidance [31]. Combined, these strategies can enhance the effectiveness of colonoscopy by promoting more consistent and adequate bowel preparation.

## 5. Conclusions

This study demonstrated key factors influencing the quality of bowel preparation for colonoscopy. Adequate bowel preparation remains the strongest predictor of a successful total colonoscopy. Age, gender, season, medication for prep, withdrawal time, and colorectal lesions were associated with bowel preparation. To improve bowel preparation quality, future studies could explore targeted interventions. These may include identifying high-risk patients and modifying patient education methods, using video presentations, and including relevant information in educational materials and protocols. The integration of a personalized approach, especially in the older population, through patient education, targeted interventions, and technological support, may be beneficial. These proposed approaches should be evaluated in future research.

## Figures and Tables

**Table 1:** Characteristics of study groups, quality bowel preparation, and total colonoscopy for the entire sample (N = 4609).

Age (years; M, SD)	62.0 (14.0)
Age groups, N (%)	
≤30 years	130 (2.8)
31–45 years	484 (10.5)
46–60 years	1250 (27.1)
61–75 years	2025 (43.9)
≥76 years	720 (15.6)
Gender, N (%)	
Female	2136 (53.7)
Male	2473 (46.3)
Season, N (%)	
Autumn	1339 (29.1)
Winter	1409 (30.6)
Spring	840 (18.2)
Summer	1021 (22.2)
Indication for colonoscopy, N (%)	
Preventive	849 (18.4)
Symptomatic	2452 (53.2)
Control	1308 (28.4)
Risk factors for colonoscopy, N (%)	
Without risk	4437 (96.3)
Anticoagulant therapy	160 (3.5)
Anticoagulant and antibiotic therapy	12 (0.3)
Day of week, N (%)	
Monday	1097 (23.8)
Tuesday	779 (16.9)
Wednesday	928 (20.1)
Thursday	917 (19.9)
Friday	888 (19.3)
Time of bowel preparation, N (%)	
Day before the exam	4303 (93.4)
Day of the exam	261 (5.7)
Day before and on the day of the exam	45 (1.0)
Preparation of bowel preparation, N (%)	
Working day	3562 (77.3)
Sunday	1047 (22.7)
Medication of bowel preparation, N (%)	
Polyethylene glycol (PEG) solution	4282 (92.9)
Senna solution	77 (1.7)
Enema	161 (3.5)
Bisacodyl	89 (1.9)
Sedation/premedication, N (%)	
None	4353 (94.4)
Yes (Propofol, Pethidine, Midazolam, antibiotic or combinations)	256 (5.6)
Irritable bowel disease, N (%)	
No irritable bowel disease	4408 (95.6)
Morbus Chron	104 (2.3)
Ulcerative colitis	97 (2.1)
Tumors, N (%)	
Yes	219 (4.8)
No	4390 (95.2)
Polyps, N (%)	
Yes	1057 (22.9)
No	3552 (77.1)
Diverticula, N (%)	
Yes	1141 (24.8)
No	3468 (75.2)
Anorectal changes, N (%)	
Hemorrhoids and other	2756 (59.8)
Without anorectal changes	1853 (40.2)
Total BBPS score, M (SD)	6.2 (2.0)
Right colon	1.7 (0.9)
Transverse colon	2.1 (0.9)
Left colon	2.4 (0.7)
Bowel preparation quality (BBPS), N (%)	
Inadequate (0–3)	626 (13.6)
Poor (4–5)	715 (15.5)
Good (6–7)	1795 (38.9)
Excellent (8–9)	1473 (32.0)
Total quality bowel preparation, N (%)	
Non-adequate (≤5)	1341 (29.1)
Adequate (≥6)	3268 (70.9)
Withdrawal time, (min; N (%))	8.7 (2.7)
Total colonoscopy, N (%)	
Yes	4050 (87.9)
No	559 (12.1)

Note: M—Mean; SD—standard deviation; N—absolute number; %—percentage; BBPS—Boston bowel preparation score.

**Table 2:** Differences in bowel preparation quality according to respondents’ characteristics (N = 4609).

	Boston Bowel Preparation	*p*-Value	Post-Hoc ^‡^
Gender			
Female	6.2 (2.0)	0.176 *	-
Male	6.3 (2.1)		
Age groups,			
≥45 years	6.7 (2.0)	<0.001 *	-
≥46 years	6.1 (2.0)		
Age groups			a/d < 0.001 a/e 0.001 b/d < 0.001 b/e < 0.001 c/d 0.006 c/e < 0.001 d/e < 0.001
18–30 years (a)	6.8 (2.2)	<0.001 ^†^	
31–45 years (b)	6.7 (1.9)		
46–60 years (c)	6.4 (1.9)		
61–75 years (d)	6.2 (2.0)		
>75 years (e)	5.6 (2.2)		
Season			
Autumn (a)	6.3 (2.0)	0.002 ^†^	sm/w 0.001
Winter (w)	6.1 (2.0)		
Spring (s)	6.2 (2.0)		
Summer (sm)	6.4 (2.1)		
Indication for colonoscopy			
Preventive (p)	6.6 (1.8)	<0.001 ^†^	p/s, p/c <0.001
Symptomatic (s)	6.1 (2.1)		
Control (c)	6.2 (2.0)		
Risk factors for colonoscopy, N (%)			
Without risk	6.2 (2.0)	0.134 ^†^	
Anticoagulant therapy	5.9 (2.1)		-
Anticoagulant and antibiotic therapy	6.3 (2.0)		
Day of week, N (%)			
Monday (m)	6.3 (2.0)	0.001 ^†^	m/f, th/f 0.002
Tuesday (t)	6.2 (2.2)		
Wednesday (w)	6.2 (2.0)		
Thursday (th)	6.4 (2.0)		
Friday (f)	6.0 (2.0)		
Procedure of bowel preparation, N (%)			
Preparation on working day	6.2 (2.1)	0.008 *	-
Preparation on Sunday	6.4 (2.0)		
Time of bowel preparation, N (%)			
the day before the exam (db)	6.3 (2.0)	<0.001 ^†^	db/ond <0.001
on the day of the exam (ond)	5.5 (2.5)		
the day before and on the day of the exam (dbe)	6.1 (2.2)		
Medication of bowel preparation, N (%)			
Polyethylene glycol solution (m)	6.4 (1.9)	<0.001 ^†^	m/e, m/b, s/e, s/b <0.001
Senna solution (s)	5.8 (2.2)		
Enema (e)	3.9 (2.3)		
Bisacodyl (b)	4.0 (2.6)		
Sedation/premedication, N (%)			
None	6.1 (2.0)	<0.001 *	-
Yes (Propofol, Pethidine, Midazolam, antibiotic or combinations)	7.2 (1.7)		
Irritable bowel disease, N (%)			
No irritable bowel disease (noi)	6.2 (2.0)	<0.001 ^†^	noi/cb; mb/uc <0.001
Morbus Chron (mc)	7.2 (1.8)		
Ulcerative colitis (uc)	5.9 (2.3)		
Tumors, N (%)			
Yes	5.2 (1.7)	<0.001 *	-
No	6.3 (2.0)		
Polyps, N (%)			
Yes	6.1 (1.9)	0.105 *	-
No	6.2 (2.0)		
Diverticula, N (%)			
Yes	6.3 (1.9)	<0.001 *	-
No	6.2 (2.1)		
Anorectal changes, N (%)			
Hemorrhoids and other	6.4 (1.9)	<0.001 *	-
Without anorectal changes	5.9 (2.2)		
Total quality bowel preparation			
Adequate (≥6)	7.3 (1.1)	<0.001 *	-
Non-adequate (≤5)	3.6 (1.3)		
Total colonoscopy			
Yes	6.6 (1.7)	<0.001 *	-
No	3.1 (1.7)		

* *t*-test, ^†^—ANOVA, ^‡^—Bonferroni post-hoc test; abbreviations in parentheses indicate the compared variables in the table.

**Table 3:** Association between bowel preparation quality and withdrawal time with patients’ characteristics using linear regression models.

	Bowel Preparation Quality (BBPS Score)			Withdrawal Time		
	β	t	*p*	β	t	*p*
Age	−0.12	−8.31	<0.001	0.00	0.20	0.840
Gender (male was referent group)						
Female	0.01	0.64	0.520	−0.02	−1.04	0.297
Indication for colonoscopy (prevention and control exams were referent group)						
Symptoms	−0.03	−1.97	0.048	0.06	4.13	<0.001
Risk factors (no risk was referent group)						
Risk yes	0.00	0.05	0.963	−0.02	−1.21	0.225
Season (summer was referent group)						
Autumn	−0.03	−1.80	0.071	0.00	0.19	0.852
Winter	−0.07	−3.99	<0.001	0.01	0.69	0.490
Spring	−0.06	−3.29	0.001	0.06	3.74	<0.001
Time of bowel preparation (day before the exam was referent group)						
On the day	0.00	0.13	0.898	−0.02	−0.65	0.513
Day before and on the day	−0.01	−0.39	0.696	−0.04	−1.15	0.251
Procedure of colon cleansing (working day was referent group)						
Cleaning on Sunday	0.03	1.85	0.064	0.04	3.07	0.002
Medications of bowel preparation (PEG solution was referent group)						
Senna solution	−0.03	−2.52	0.012	−0.01	−0.99	0.321
Enema	−0.20	−12.93	<0.001	−0.01	−0.43	0.665
Bisacodyl	−0.16	−11.15	<0.001	−0.08	−5.20	<0.001
Premedication or sedation (no was referent group)						
Yes	0.09	6.56	<0.001	0.00	0.06	0.952
Irritable bowel disease (no was referent group)						
Morbus Chron	0.06	3.89	<0.001	−0.02	−1.63	0.103
Ulcerative colitis	−0.01	−0.97	0.332	−0.03	−2.22	0.026
Tumor (no was referent group)						
Tumor (yes)	−0.06	−4.07	<0.001	−0.02	−1.22	0.222
Polyp (no was referent group)						
Polyp (yes)	−0.02	−1.46	0.145	0.12	8.10	<0.001
Diverticula (no was referent group)						
Diverticula (yes)	0.08	5.35	<0.001	−0.01	−0.80	0.423
Anorectal changes (no was referent group)						
Anorectal changes (yes)	0.10	7.29	<0.001	−0.19	−13.51	<0.001
Bowel preparation quality (BBPS)	-	-	-	0.09	5.69	<0.001
Withdrawal time, (min)	0.08	5.69	<0.001	-	-	-

Note: BBPS—Boston bowel preparation score, β—beta coefficient, t—t statistics, *p*—*p* value.

**Table 4:** Associations of adequate bowel preparation and complete total colonoscopy using logistic regression models (N = 4609).

	Adequate Bowel Preparation			Total Colonoscopy		
	OR	95% CI	*p*	OR	95% CI	*p*
Age (years)	0.98	(0.97–0.99)	<0.001	0.99	(0.98–1.00)	0.041
Gender (male was referent group)						
Female	1.18	(1.01–1.37)	0.038	0.71	(0.57–0.90)	0.004
Indication for colonoscopy (prevention and control exams were referent group)						
Symptoms	1.05	(0.90–1.23)	0.499	0.54	(0.42–0.68)	<0.001
Risk factors (no risk was referent group)						
Risk yes	1.14	(0.77–1.68)	0.523	0.78	(0.44–1.36)	0.377
Season (summer was referent group)						
Autumn	0.96	(0.77–1.19)	0.711	1.05	(0.76–1.46)	0.748
Winter	0.74	(0.60–0.91)	0.004	1.07	(0.78–1.46)	0.682
Spring	0.92	(0.72–1.16)	0.474	0.97	(0.68–1.40)	0.885
Time of bowel preparation (day before the exam was referent group)						
On the day	1.22	(0.84–1.77)	0.305	0.97	(0.59–1.58	0.890
Day before and on the day	0.92	(0.42–2.02)	0.845	0.86	(0.29–2.52)	0.787
Procedure of colon cleansing (the working day was referent group)						
Cleaning on Sunday	1.09	(0.91–1.30)	0.363	1.26	(0.94–1.68)	0.116
Medications of bowel preparation (polyethylene glycol was referent group)						
Senna solution	0.57	(0.34–0.96)	0.035	1.41	(0.61–3.26)	0.425
Enema	0.22	(0.14–0.36)	<0.001	0.36	(0.22–0.58)	<0.001
Bisacodyl	0.35	(0.20–0.61)	<0.001	0.24	(0.13–0.45)	<0.001
Premedication or sedation (no was referent group)						
Yes	2.29	(1.49–3.53)	<0.001	1.10	(0.58–2.10)	0.776
Irritable bowel disease (no was referent group)						
Morbus Chron	1.48	(0.81–2.70)	0.205	2.44	(0.83–7.15)	0.105
Ulcerative colitis	1.23	(0.70–2.16)	0.463	0.44	(0.22–0.90)	0.025
Tumor (no was referent group)						
Tumor (yes)	1.33	(0.91–1.95)	0.142	0.22	(0.14–0.34)	<0.001
Polyp (no was referent group)						
Polyp (yes)	0.87	(0.73–1.04)	0.124	1.43	(1.06–1.91)	0.018
Diverticula (no was referent group)						
Diverticula (yes)	1.51	(1.25–1.81)	<0.001	0.98	(0.74–1.29)	0.872
Anorectal changes (no was referent group)						
Anorectal changes (yes)	1.54	(1.32–1.79)	<0.001	1.07	(0.85–1.36)	0.542
Withdrawal time, (min)	0.94	(0.92–0.97)	<0.001	1.27	(1.22–1.33)	<0.001
Adequate BPPS *	-	-	-	23.60	(17.84–31.22)	<0.001
Total colonoscopy	27.91	(20.96–37.16)	<0.001	-	-	-

Note: OR—odds ratio; 95% CI—95% confidence interval, *p*—*p*-value, * BBPS—Boston bowel preparation score ≥ 6.

## Data Availability

The dataset analyzed in this study is available from the corresponding author upon request.

## Supplementary Materials

The following supporting information can be downloaded at: https://www.mdpi.com/article/10.3390/medicina62010063/s1, Supplemental Table S1: Differences by gender, age group, season, day of the week, and bowel preparation solution in the category of quality of bowel preparation (N = 4609).

## Abbreviations

The following abbreviations are used in this manuscript:

AI	artificial intelligence
BBPS	Boston bowel preparation score
CI	confidence interval
CRC	colorectal cancer
ECIS	European Cancer Information System
GI	Gastro intestinal
OR	odds ratio
PCCR	Post Colonoscopy Colorectal Cancer
PEG	polyethylene glycol
RG	referent group
