# Supplementary material for: Quality of Bowel Preparation in the General Population
Source: Medicina (Kaunas). 2025 Dec 28;62(1):63. doi: 10.3390/medicina62010063 (PMC12843468; doi:10.3390/medicina62010063)
Supplement: Supplementary file 1 [file medicina-62-00063-s001.zip › medicina-3998829-supplementary.pdf]

Supplemental Table S1. Differences by gender, age group, season, day of the week, and bowel preparation solution in the category of quality of bowel preparation (N=4609)

	Inadequate (0–3)	Poor (4–5)	Good (6–7)	Excellent (8–9)	p-value
Gender					
Male	322 (51,4)	416 (58,2)	979 (54,5)	756 (51,3)	0.012
Female	304 (48,6)	299 (41,8)	816 (45,5)	717 (48,7)	
Age group					
≤ 30	21 (3,4)	6 (0,8)	36 (2,0)	67 (4,5)	<0.001
31-45	45 (7,2)	51 (7,1)	187 (10,4)	201 (13,6)	
46-60	139 (22,2)	170 (23,8)	517 (28,8)	424 (28,8)	
61-75	275 (43,9)	341 (47,7)	800 (44,6)	609 (41,3)	
≥ 76	146 (23,3)	147 (20,6)	255 (14,2)	172 (11,7)	
Seasons					
Autumn	166 (26,5)	206 (28,8)	554 (30,9)	413 (28,0)	<0.001
Winter	209 (33,4)	245 (34,3)	536 (29,9)	419 (28,4)	
Spring	112 (17,9)	125 (17,5)	355 (19,8)	248 (16,8)	
Summer	139 (22,2)	139 (19,4)	350 (19,5)	393 (26,7)	
Day of week, N (%)					
Monday	141 (22,5)	164 (22,9)	409 (22,8)	383 (26,0)	0.001
Tuesday	117 (18,7)	119 (16,6)	276 (15,4)	267 (18,1)	
Wednesday	112 (17,9)	153 (21,4)	396 (22,1)	267 (18,1)	
Thursday	111 (17,7)	135 (18,9)	354 (19,7)	317 (21,5)	
Friday	145 (23,2)	144 (20,1)	360 (20,1)	239 (16,2)	
Medication of bowel preparation					
Moviprep (generičko ime?)	475 (75,9)	659 (92,2)	1723 (96,0)	1425 (96,7)	<0.001
Xprep (generičko ime?)	17 (2,7)	12 (1,7)	27 (1,5)	21 (1,4)	
Enema	85 (13,6)	34 (4,8)	291,6)	13 (0,9)	
Bisacodyl	49 (7,8)	10 (1,4)	16 (0,9)	14 (1,0)	

Note:p-p-value
